# Early Identification of Maternal Cardiovascular Risk Through Sourcing and Preparing Electronic Health Record Data: Machine Learning Study

**DOI:** 10.2196/34932

**Published:** 2022-02-10

**Authors:** Nawar Shara, Kelley M Anderson, Noor Falah, Maryam F Ahmad, Darya Tavazoei, Justin M Hughes, Bethany Talmadge, Samantha Crovatt, Ramon Dempers

**Affiliations:** 1 MedStar Health Research Institute Georgetown-Howard Universities Center for Clinical and Translational Science Hyattsville, MD United States; 2 Georgetown University Washington, DC United States; 3 MedStar Health Research Institute Hyattsville, MD United States; 4 Invaryant Inc Roswell, GA United States

**Keywords:** electronic health record, maternal health, machine learning, maternal morbidity and mortality, cardiovascular risk, data transformation, extract, transform, load, artificial intelligence, electronic medical record

## Abstract

**Background:**

Health care data are fragmenting as patients seek care from diverse sources. Consequently, patient care is negatively impacted by disparate health records. Machine learning (ML) offers a disruptive force in its ability to inform and improve patient care and outcomes. However, the differences that exist in each individual’s health records, combined with the lack of health data standards, in addition to systemic issues that render the data unreliable and that fail to create a single view of each patient, create challenges for ML. Although these problems exist throughout health care, they are especially prevalent within maternal health and exacerbate the maternal morbidity and mortality crisis in the United States.

**Objective:**

This study aims to demonstrate that patient records extracted from the electronic health records (EHRs) of a large tertiary health care system can be made actionable for the goal of effectively using ML to identify maternal cardiovascular risk before evidence of diagnosis or intervention within the patient’s record. Maternal patient records were extracted from the EHRs of a large tertiary health care system and made into patient-specific, complete data sets through a systematic method.

**Methods:**

We outline the effort that was required to define the specifications of the computational systems, the data set, and access to relevant systems, while ensuring that data security, privacy laws, and policies were met. Data acquisition included the concatenation, anonymization, and normalization of health data across multiple EHRs in preparation for their use by a proprietary risk stratification algorithm designed to establish patient-specific baselines to identify and establish cardiovascular risk based on deviations from the patient’s baselines to inform early interventions.

**Results:**

Patient records can be made actionable for the goal of effectively using ML, specifically to identify cardiovascular risk in pregnant patients.

**Conclusions:**

Upon acquiring data, including their concatenation, anonymization, and normalization across multiple EHRs, the use of an ML-based tool can provide early identification of cardiovascular risk in pregnant patients.

## Introduction

### Background

Each year in the United States, maternal morbidity and mortality (MMM) accounts for more than 700 deaths and an additional 50,000 life-threatening complications associated with pregnancy and childbirth [1]. It is estimated that 70% of these events are preventable [1]. Cardiovascular disease accounts for 60% of maternal morbidity events and over one-third of maternal deaths in the United States [2]. More than 50% of MMM events are attributed to cardiovascular causes including cardiomyopathy (11.5%), thrombotic pulmonary embolism (9.6%), cerebrovascular accidents (8.2%), hypertensive disorders of pregnancy (6.6%), and other cardiovascular conditions (15.5%) [1]. In 2019, the national maternal death rate was 20.1 deaths per 100,000 live births [3]. It is estimated that 68.2% of pregnancy-related cardiovascular deaths are preventable [4].

Exacerbating the crisis, health care data are fragmented as patients seek care from diverse sources, including different health care systems and telehealth providers. Consequently, coordinating patients’ care with disparate health records continues to increase in complexity.

We hypothesize that a systematic method for identifying risk early by analyzing changes in a patient’s health data based on complete data set trends is possible and facilitates early intervention and treatment of high-risk conditions in pregnant women. Early identification and intervention of these conditions would likely result in a measurable reduction in maternal fatalities and life-threatening complications.

A previous study focused on predicting common maternal postpartum complications by leveraging machine learning (ML) and electronic health records (EHRs) highlighted the risk level of maternal postpartum complications requiring inpatient care [5]. Data were gathered from patients’ dates of gestation to delivery and demonstrated that routinely collected health data, when used in conjunction with ML, have the potential to accurately predict postpartum outcomes [5].

With this as our basis, our aim is to demonstrate that ML and aggregated EHRs can be leveraged to surface signals and trends in patients’ medical records to identify predictors of cardiovascular conditions during pregnancy.

Through a retrospective study based on a cohort from a large tertiary health care system of 32,409 patients who were seen during pregnancy, we demonstrate that Invaryant’s Health Outcomes for all Pregnancy Experiences–Cardiovascular-Risk Assessment Technology (HOPE-CAT), an ML-based risk assessment algorithm, identifies factors that may indicate the development of cardiovascular conditions that lead to MMM.

### Overview—Data and ML

ML is becoming a disruptive force in health care, and its application is broad, including imaging, risk identification, and risk assessment to inform and improve patient care and outcomes [6]. Recent studies have demonstrated that ML, compared with traditional statistical modeling, is a more effective tool in predicting sex-specific and cardiovascular diseases [7]. In addition, when combined with traditional logical regression, ML may assist in identifying novel predictors of disease [8].

However, the vast differences that exist in each individual’s corpus of health data and the lack of standards to define the capture of data create challenges for ML. In addition to complexity and variation among patients, there are systemic issues that render the data unreliable and fail to create a single accurate view of each patient. Despite standardization efforts, including Fast Healthcare Interoperability Resources and Continuation of Care Documents, adoption of these standards and upgrades is slow. In addition, within health care, patients often receive care from different providers and specialists for various conditions, obtaining diverse medications and treatments without a *clearinghouse* to ensure that all providers have access to all relevant data. Finally, owing to lack of standardization, when data are sourced from disparate systems, the resulting data must be cleaned and normalized to be made actionable. The lack of connectivity in health care creates challenges for providers, who provide care with limited and often incomplete patient information.

It is important to note that for this study, all available data were sourced from a single health care system with 10 hospitals in addition to outpatient clinics, which was both an advantage and a challenge. One advantage was that the data set represented a diverse patient population in a system with many hospitals and outpatient clinics. However, because there was only access to the single system, if a patient sought care at an external facility, the data from those visits were not available in the data set. This demonstrates one of the major challenges of the aforementioned lack of interconnectivity within the US health care system.

Despite the defined systemic problems, ML models have several advantages for the assimilation and evaluation of complex health care data. Unlike traditional statistical models, ML offers flexibility and scalability, which makes it deployable for many tasks, such as risk stratification, diagnosis and classification, and survival intervention [9]. However, when considering the use of these tools for health care data, one must understand that there are limitations to be anticipated and considered. Ethically speaking, notwithstanding the systemic issues described, clinical implementation of the technology must be for the direct benefit of a patient and their providers. The completeness of data cannot be assured, nor can it be assumed that those data are always accurately captured; additionally, the ethical use of these technologies mandates respect for patients’ sensitive personal health information throughout their use.

## Methods

### Technology

For this project, the following software and platforms were used: 4 Cerner Millennium (edition 2018.01) electronic medical record software, PeriBirth (PeriGen), R (version 4.05; R Foundation for Statistical Computing); Microsoft Azure Cloud, Microsoft Azure Data Studio, Microsoft Azure Machine Learning Studio, virtual machine, Microsoft SQL Server Management Studio, Invaryant’s health platform, and HOPE-CAT.

For the purpose of this study, HOPE-CAT analyzes an individual patient’s EHR data on an encounter-by-encounter basis to identify risk factors (eg, elevated blood pressure readings, shortness of breath, and chest pain) indicative of the development or worsening of cardiovascular conditions.

HOPE-CAT was trained via causal inference, with limited training supervision, using established maternal cardiovascular risk factors and covariates, such as physical findings, symptoms, and medical history (Textbox 1).

**Risk factors and covariates used to train the Health Outcomes for all Pregnancy Experiences–Cardiovascular-Risk Assessment Technology.**

- **Symptoms (variable risks)**
  Dyspnea (red flag risk)
  Orthopnea (red flag risk)
  Tachypnea
  Asthma unresponsive to therapy
  Swelling in face or hands
  New or worsening headache
  Heart palpitations
  Dizziness or syncope
  Chest pain
- **Physical findings (variable risks)**
  Loud heart murmur
  Basilar crackles in lungs
  Resting heart rate≥120 beats per minute (red flag risk)≥110 beats per minute
  Systolic blood pressure ≥160 mm Hg (red flag risk)≥140 mm Hg
  Respiratory rate ≥30 (red flag risk)≥24
  Oxygen saturation ≤94% (red flag risk)≤96%
- **Medical history (static risks)**
  Aged ≥40 years
  Race=African American
  Prepregnancy obesity (BMI≥35)
  Prepregnancy diagnosis of diabetes
  Prepregnancy diagnosis of hypertension
  Substance use (nicotine, cocaine, alcohol, and methamphetamines)
  History of chemotherapy
  History of complications in labor or delivery
  History of heart disease

HOPE-CAT was then used to simulate chronological patient encounters as they occurred in the medical records. The onsets of risk detected by HOPE-CAT were compared with EHR-recorded diagnoses or interventions in the source data’s timeline. Loss vectoring methods were used to determine the delta, or difference, between HOPE-CAT’s outputs and the anticipated outputs, thereby guiding the learning and training. In this study, the patient encounters and outcomes were already known, and HOPE-CAT was configured to simulate patient encounters (eg, clinic appointments, emergency department visits, and hospital admissions) on the encounter dates recorded in each patient’s EHR to assess the available data and detect potential risks. A delta was then determined between HOPE-CAT’s assessments and actions taken by the health care provider on the same dates with the same information.

### Criteria and Requirements Assessment

Inclusion and exclusion criteria to be pulled from the EHR were defined to create the data set to ensure that the algorithms had adequate data to analyze for trends and were able to designate risk profiles as early in the process as possible for each patient. Inclusion and exclusion criteria were agreed upon by clinical cardiovascular and maternal health experts and data scientists. These inclusion criteria included patient demographics (eg, age, race, and geographic location), physiologic measures (eg, blood pressure, heart rate, and oxygen saturation), symptoms (eg, headache and shortness of breath), and health history from each patient encounter during pregnancy. Once criteria were defined, the list of variables were organized to identify the sources of data required.

### Data Sourcing, Cleansing, Scrubbing, and Normalization

#### Data Acquisition (Institutional Policy Compliance)

For this study, institutional privacy and security policies were followed to ensure that patient data were protected and secure throughout the project. Institutional review board approval was obtained from the study institution. Analysts handling the data maintain standing access to various databases containing patient-EHR and other research data. Access is individualized and maintained through the institution’s active directory. All activities within these systems are tracked and auditable, and institutional review board approval is required before any research-related data extraction. Data access methods, as well as data extraction, transfer, and anonymization procedures, were reviewed by the institution’s data security team before the creation of the shared analysis environment to ensure all necessary security requirements were implemented before the release of data.

#### Data Sources and Extraction

There were multiple data sources within the hospital system, each with its own access restrictions, and in some cases, data sources were administered by different departments or groups within the hospital. The 2 primary systems used were a direct connection to Cerner’s underlying Oracle database, as well as an enterprise data warehouse (EDW) solution, which contains data from the Cerner EHR, as well as other third-party billing, quality, and safety systems employed at the different member institutions. Access to both systems was controlled through specific roles defined in the active directory, the Microsoft Lightweight Directory Access Protocol service. RStudio (running R version 4.05) was used to query both data sources for extraction, as well as for subsequent data transformation. It should be noted that it is theoretically possible to perform discovery and extraction of this data using Cerner’s supplied suite of tools (eg, Discern Analytics and Cerner Command Language); however, because of the large number of variables and size of the data set, having other solutions available provided a significant advantage, both in performance and ease of use.

Both EDW and Cerner use a relational database architecture. Cerner’s data model is primarily *visit-centric*, which means that most data created within the EHR tie together via a unique encounter ID that is created for each visit. Visits connect together through a unique person ID, and certain tables—such as the address table, family history table, and problems table (for chronic conditions)—are kept at the *person* grain. The EDW keeps these source identifiers and also includes additional fields to allow for cross-walking of visits and patients between the different imported data sources.

The data selection process began with a baseline population of patients who had a documented delivery between January 1, 2017, and December 31, 2020. A delivery was defined as a documented delivery procedure as outlined by the Centers for Disease Control and Prevention [1]. From the initial population, International Classification of Diseases, 10th Revision (ICD-10), diagnosis codes were used to identify those patients’ prenatal visits. After compiling the initial list of visits for each patient, diagnoses, selected clinical variables, and personal information (demographics) were abstracted for each patient.

In addition, visits created because of a historical upload or import from another source were excluded. These visits had registration dates starting in 1900. An age filter was also implemented so that only data from patients aged 18-35 years at the time of the encounter were received. *Visit* entries that were created because of communication between staff and patients, such as patient portal messages or phone calls, were removed if there was no relevant clinical data or if the data otherwise did not meet the established inclusion criteria.

The EDW was used for supplemental data not housed within the main EHR environment, such as diagnosis-related group codes to categorize diagnoses and complications, as well as *cleaned* versions (with duplicates removed) of certain types of data, such as medication administrations, to prevent duplicate work. A large portion of the clinical data needed, such as laboratory results, measurements, and other discrete clinical observations, were sourced from the *clinical events* table within Cerner and further categorized after extraction. The clinical events table uses the same field for result values regardless of the variable, so additional fields, such as the result *unit* (eg, lb, kg, and mm Hg), were included for additional context; this also allowed for the comparison of variables, such as weight, that can be entered as either pounds or kilograms.

This time-consuming exercise was simplified by the creation and maintenance of a comprehensive, well-documented data catalog using the tools provided in the database administration studio, which was updated when data were added from the source systems (ie, the metadata repository or data dictionary). Cerner does provide a table that has some preconfigured event categorizations in a hierarchy. However, the hierarchies and category labels are customizable at each institution, so manual review was still required to create comprehensive groups.

Validation checks were completed by manually combing through the events list to check if any code or piece of information had been missed. This step was crucial for maternal history and delivery information, as these can be documented in different ways because of various workflows across different hospitals or departments. Free-text clinical notes were not used for this study because of the additional time and computing resources that would have been required for proper removal of protected health information and identification of clinically relevant text.

A series of checks were also completed to ensure the accuracy of the data. After a variable list was developed, the data were once again validated to ensure that the variables were accounted for within the events. Randomized individual visits were then selected to check for events that would be relevant and were not already in a categorized set.

#### Data Anonymization

Although the data were administratively and medically permitted to be viewed, for privacy, they were deidentified, and therefore, variables were transformed in stages before being transferred into the dedicated Azure environment for analysis. The first set of variables transformed were basic demographic information, such as patient name and address. Zip codes were compared with census data and discarded after characterization as urban, rural, or suburban. Date time fields, such as the registration date time for visits and date time stamps associated with visits, were split into 2 fields containing the date and time of the event, respectively. The date of the index visit was calculated for each unique patient, and the date field was transformed to represent the number of calendar days from that index visit date. Time fields were kept for sequencing of events within a patient’s course. Within the system, the unique person and encounter IDs were hashed to create new person and visit IDs to prevent reidentification. A master key was created to tie patients and visits together and was only available locally to the data analysts at the home institution. The key could also be used to backtrack and revalidate in the case of errors if something did not make logical sense or if additional variables needed to be pulled after the original extraction.

The last piece of deidentification involved the events themselves. This involved parsing procedural histories and removing event types, such as comments, dates, and other free-text entry fields where identifying patient information could potentially be entered.

#### Data Transformation

After completion of the initial extraction and anonymization of the data, a new database schema was necessary to house and store the results for subsequent analysis. As a result, additional data transformation was necessary to combine different but related data elements into a single table and to aid HOPE-CAT. As stated previously, a large portion of the clinical data were further categorized after extraction. Three new fields were created to accomplish this, based on the categorization of the data fields: CATEGORY, SUB_CATEGORY, and CLINICAL_CAT. The field CATEGORY is the parent hierarchy, consists of values such as *event* and *diagnosis*, and signifies from which set of tables the event came. SUB_CATEGORY is the next level down and changes based on context. In the diagnosis table, example values include *admitting diagnosis* and *discharge diagnosis*, whereas examples from the *events* table include *labs*, *measurements*, and *medication administration*. CLINICAL_CAT is not used in all tables but provides additional categories, such as *blood gases*, *metabolic panel*, *infectious disease*, and *hematology* for lab events and *vitals* and *weight and BMI* for measurements. These 3 fields helped standardize data in different tables for easier processing.

**Figure 1:**
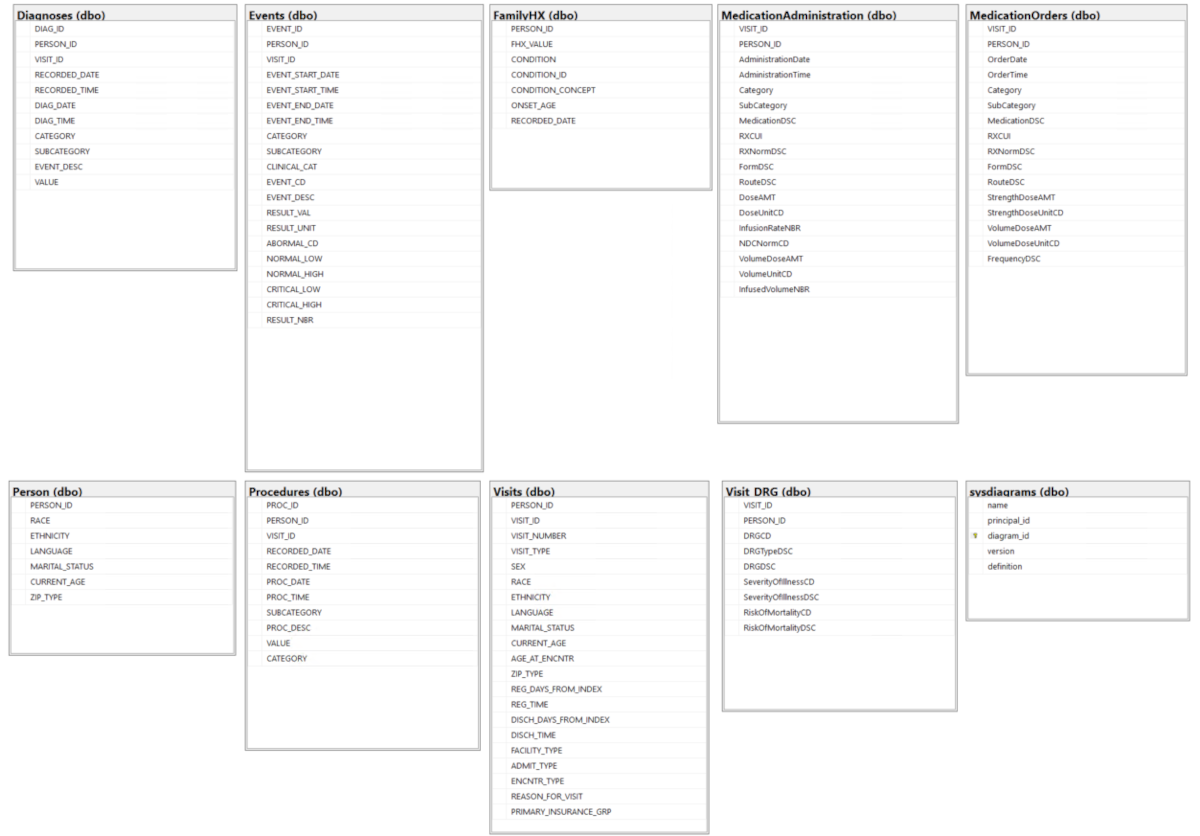
Database schema.

The final database schema used a relational structure similar to that of the original tables in the EHR. All of the final tables, with the exception of the FamilyHX and Race tables, contained both the transformed person IDs and the transformed visit IDs to allow for easier analysis at either the visit or patient level. The final database schema is shown in Figure 1.

#### Normalizing and Loading

Data were provided to Invaryant’s team in the form of a Microsoft SQL Server database, hosted on a Microsoft Azure Virtual Machine. A data catalog was created to provide the team with an inventory of available data. In addition to data profiling, statistics, and other contents, the data catalog also provided a descriptive index pointing to the location and type of available data. Owing to the large volume of data, the tables were individually loaded to the database, using Azure Data Studio, as CSV files (Table 1). Indexes were later added to the tables on key fields to make queries necessary for analysis optimization.

**Table 1:** Database overview.

Description			Value, n (%)
Total patients in the database			32,409 (100)
Patients with at least one risk identified			18,095 (55.83)
Patients who delivered on the first visit^a^			14,855 (45.83)
Patients who only had 1 visit			11,485 (35.44)
Patients with *red flag* risk levels identified^b^			1716 (5.29)
**Number of births**			
	Total births	37,457 (100)	
	Single live births	36,564 (97.62)	
	Twin births	545 (1.45)	
	Triplet births	13 (0.03)	
	Stillbirths	294 (0.78)	
**Number of patients in top detected conditions**			
	Preeclampsia	3468 (10.7)	
	Eclampsia	29 (0.09)	
	Cardiomyopathy	34 (0.1)	
	Cerebral infarction (stroke)	5 (0.02)	
**Number of patients with static risks based on category**			
	BMI≥35	2800 (8.64)	
	African American	8194 (25.28)	
	History of substance use	3469 (10.7)	

^a^These were excluded as there was no supporting retrospective data.

^b^Specific severe risk factors or 4 or more total risk factors.

### ML Training and Execution

#### Training Networks and Building Layers

To validate HOPE-CAT against retrospective patient records through simulated patient encounters (ie, office visits) from the data, training was first completed. HOPE-CAT was trained to assess the available data chronologically by visit, as providers would have recorded them in real time. To account for the anonymization of patient-encounter dates, HOPE-CAT was trained to work using a duration function (day count), rather than a date function, to accurately determine the delta. Data collected at, and related to, each visit (eg, patient demographics, physical findings, symptoms, and medical history) were provided as input to HOPE-CAT for analysis to detect changes and trends in the patient’s data. If HOPE-CAT detected risk based on the visit data and the risk factors in which it was trained (Textbox 1), a risk profile was generated for that specific patient encounter. Two types of risk profiles were generated indicating standard risk or high risk, noted as a *red flag*. Red flag risks indicated that the patient was experiencing either single severe physiological symptoms (eg, elevated blood pressure or orthopnea) or multiple risk factors (4 or more) that may be predictors of needing immediate evaluation. A risk profile establishes that risk factors indicative of the development of severe or worsening cardiovascular conditions are present. These conditions include, but are not limited to, preeclampsia, eclampsia, peripartum cardiomyopathy, cerebral infarction, myocardial infarction, heart failure, and pulmonary embolism.

Typically, HOPE-CAT evaluates for a patient’s individual baseline metrics before further analysis. For example, if a patient’s systolic blood pressure baseline is lower than the medical mean, a high reading would be below the medically recommended high-risk value in cases such as preeclampsia. However, owing to the nature of retrospective data and, in many cases, the lack of medical history, establishing personal baselines for each patient was disabled for this study. Therefore, any patient whose data had fallen outside the medically accepted averages (norms) was flagged and not used for training.

Refining the data ingested during data preparation allowed for the isolation of data that related directly to patients who exhibited static risk (ie, patient information that does not change, such as race and prepregnancy history) or variable risk factors (eg, physiologic measures and symptoms). The networks were trained to identify both an individual’s static risk factors and any additional variable risk factors developed over time. Throughout the training process, Periodic testing was performed. For missing data (eg, weight), those data were requested from the data sources and added to the study database, and the catalog and data dictionaries were updated. Once each issue was resolved, the ingestion and refining process was continued, and testing was repeated before additional training.

The training results were reviewed by clinical experts, and some adjustments were made in the context of *static* risk. The system was retrained to accommodate these changes, and once again, a series of manual tests were run to ensure that the changes had the appropriate effect.

As data were layered into the HOPE-CAT, outliers or patients with data that did not meet the evaluation criteria (eg, a single visit encounter was available, meaning trends could not be identified) were identified and flagged for exclusion (Table 1). It was found that certain data layers initially included in the requirements had limited use and that some of the data were held in other tables and, in some cases, in other databases.

#### Testing ML Outputs

Test data based on the specified inclusion criteria from cardiovascular and maternal health experts, and findings of previous studies, were used to train HOPE-CAT using human reviews of maternal data. These metrics and parameters were loaded and run against the test data. The outputs from the test data set were reviewed manually on a patient-by-patient basis. The advantage this study had in the context of medical care is that the retrospective data had clearly defined outcomes for all the patients included in the result set, thereby allowing precise analysis of HOPE-CAT’s outputs, with direct confirmations of the correlation of the defined risk to the outcome of the pregnancy. For a risk coded or identified by HOPE-CAT, it was possible to determine the accuracy of the assessment against hard data (eg, the patient being diagnosed with a cardiovascular condition).

#### Running ML and Reviewing Results

Once training was complete, HOPE-CAT was run against the full data set to determine the risk level against the encounter-duration function. When HOPE-CAT identified a certain level of risk, the encounter date associated with the output was compared with the date of when a diagnosis was made or the provider intervened (ie, the delta). The delta between the detection by HOPE-CAT and the diagnosis or intervention by the provider was assessed and quantified. In most cases, HOPE-CAT had the advantage over the provider as HOPE-CAT had a single, condensed view to the patient’s historical data, data trends, and micro and macro changes in the patient’s health. As described earlier, the advantage of the retrospective data allowed for in-depth manual reviews of the data. The process involved reviewing each method by retrieving the relevant data against the results of HOPE-CAT, and each result was cross-checked and tabulated. The tabulated information was then cross-checked by the independent quality team. An important part of ML is the classification of outputs, which identifies errors or artifacts that the system cannot explain. These data were flagged for human review and classification; as the system was designed to detect primarily cardiac-related events, it did not know how to classify certain events; therefore, HOPE-CAT flagged them as errors. Once reviewed by the data analysts, a set of these errors were identified as *organ failures*, and in review of the data, all references to organ failure in the data were detected, and the classification was added to the classification system. This resulted in the expansion of the classification algorithm to alert providers of the additional risk of potential organ failure in a patient, indicating that a patient may require further monitoring and intervention to prevent advancement of disease state and more severe outcomes. For example, patients with HELLP (hemolysis, elevated liver enzymes, and low platelets) syndrome or preeclampsia should be monitored for hematologic changes or changes in liver or kidney function, respectively, which may indicate disease advancement and potential organ failure. This process demonstrates that error handling is an effective tool for identifying and correcting omissions or unexpected events in the data.

## Results

This study has shown that patient records from EHRs, when aggregated, can be made actionable for the goal of effectively using ML, specifically to identify cardiovascular risk in pregnant patients. The resulting delta informs future studies in which HOPE-CAT will be deployed to monitor for and alert providers to real time trends in patient data.

## Discussion

### Limitations

Several methods used within this study are proprietary to Invaryant. These methods are related to HOPE-CAT ML, the risk stratification algorithm designed to establish patient-specific baselines to identify and establish cardiovascular risk based on deviations from the patient’s baseline. That said, these processes being proprietary to Invaryant do not limit future research in this purview. ML processes similar to HOPE-CAT may be developed; however, the processes of training, variable weighting, and validating may differ.

### Conclusions

Within this study, 32,409 anonymized health records were extracted from multiple Cerner EHR systems. Data were collected and applied in four distinct steps: design, discovery, ingestion, and refinement. Extensive measures were taken to meet patient privacy requirements and the home institution’s security requirements, including removing key identifiable data points, including names, addresses, dates of birth, and zip codes, as well as other measures to protect patient privacy. Further security measures were taken to provide access to the data and establish the environment in the Microsoft Azure Cloud while maintaining the home institution’s security policies and practices. Data were then cleaned, scrubbed, validated, structured, optimized, and normalized before setting up analytical processing capabilities. To prepare for analytical processing, iterative, layered training of samples of the data was executed, and reviewed training for the learning engine was run, to ensure an abundance of data categories were available in large enough quantities to guarantee that results were reproducible and scalable for complete analysis in a *real-world* live setting. The latter part will be vital in instances where these processes are used in vivo.

Future studies involving the HOPE-CAT may include the following: the addition of geographic data and other data related to social determinants of health, including unstructured sources (eg, chart notes, family histories, and imaging) with natural language processing or prospective in vivo application.

### Recommendations From This Study and for Future Studies

During the design process, it is recommended to consider the following:

- Are enough data available to represent the pattern of interest?
- Are the data available accurate? (Plausibility checks for accuracy, misspellings, parsing, and standardization to specific locales are recommended.)
- Have data correction schemes been considered?
- Data-cleaning decisions go beyond technical feasibility; evaluating ethical and legal implications is also necessary.
- Including an iterative review process with clinicians for algorithm-inclusion requirements is recommended.
- Educating end users on the many implications (medical, legal, and ethical) of using these technologies to inform better health outcomes and setting expectations for artificial intelligence and ML strengths and limitations are recommended.
- Training end users on how best to use artificial intelligence and ML tools and interpret outputs is recommended.

Before gathering data, source selection is key. It is important to first determine if data will need to be gathered from multiple sources, and if so, how to integrate them. Assessing the number of events required per observation period and determining beforehand how much data are needed to represent segment variability or to simply come to a successful conclusion could be very useful. Formulating an easy method for matching data from alternate sources is key to ensuring sufficient data for any project.

Data preparation methods should include formal processes, such as the creation of dictionaries, catalogs, and other controls, that allow the process to be repeatable and scalable. Metadata, persistent managed storage, and reusable transformation or cleansing, and the information around them, must be included to make data preparation efficient and consistent. Assessing how the data need to be aligned for the analysis often involves cardinality, binning, correlations, derivations of new values, gender or identity analyses, and other methods to prepare data at the needed level of granularity. Within this study, it was found that once data refinement was started, additional data were needed to better suit the purpose of the study. During the process of refining data, it is recommended that one determines how fit the data are for the intended purpose and if further data may be needed.

## Abbreviations

EDW: enterprise data warehouse
EHR: electronic health record
HELLP: hemolysis, elevated liver enzymes, and low platelets
HOPE-CAT: Health Outcomes for all Pregnancy Experiences–Cardiovascular-Risk Assessment Technology
ICD-10: International Classification of Diseases, 10th Revision
ML: machine learning
MMM: maternal morbidity and mortality
